# Fast and accurate protein substructure searching with simulated annealing and GPUs

**DOI:** 10.1186/1471-2105-11-446

**Published:** 2010-09-03

**Authors:** Alex D Stivala, Peter J Stuckey, Anthony I Wirth

**Affiliations:** 1 Department of Computer Science and Software Engineering, The University of Melbourne, Victoria 3010, Australia; 2 National ICT Australia Victoria Laboratory at The University of Melbourne, Victoria 3010, Australia

## Abstract

**Background:**

Searching a database of protein structures for matches to a query structure, or occurrences of a structural motif, is an important task in structural biology and bioinformatics. While there are many existing methods for structural similarity searching, faster and more accurate approaches are still required, and few current methods are capable of substructure (motif) searching.

**Results:**

We developed an improved heuristic for tableau-based protein structure and substructure searching using simulated annealing, that is as fast or faster and comparable in accuracy, with some widely used existing methods. Furthermore, we created a parallel implementation on a modern graphics processing unit (GPU).

**Conclusions:**

The GPU implementation achieves up to 34 times speedup over the CPU implementation of tableau-based structure search with simulated annealing, making it one of the fastest available methods. To the best of our knowledge, this is the first application of a GPU to the protein structural search problem.

## Background

Searching a database of protein structures for structures that are similar to, or contain substructures that are similar to, a query structure is a significant problem in structural biology and bioinformatics. We can classify methods for protein structural searches into four categories. First, methods that align proteins directly at the level of residues. Dali and DaliLite [1,2] fall into this category. Second, methods that align proteins at the level of secondary structure elements (SSEs). TableauSearch [3], ProSMoS [4], and the TOPS-based methods [5,6] fall into this category. Third, methods that perform an initial alignment at the level of SSEs, and then extend it to a residue level alignment. VAST [7,8], SSM [9], LOCK2 [10], and SARF2 [11] fall into this category. Fourth, methods that do not perform an alignment at all, but use some other means of providing a similarity score. YAKUSA [12] and PRIDE [13-15] fall into this category. Methods in the first category tend to be the slowest, since they are not necessarily designed solely or primarily for database scanning, but also to provide a set of correspondences between residues. Since the number of residues is naturally much larger than the number of SSEs, these methods must solve problems of a larger size than SSE-based methods.

SHEBA [16] and YAKUSA both use a one-dimensional representation of protein structure to accelerate structural searching. SHEBA uses "environmental profiles" containing information about sequence homology and residue-dependent information such as solvent accessibility, hydrogen bonds, and side-chain packing, which is then refined for three-dimensional geometry by dynamic programming. YAKUSA is a fast method that uses a one-dimensional representation based on protein internal angles.

We note that the classification just described is not strict or even exclusive. For example, we place SHEBA in the first category since it provides a residue-level alignment, but it does not do so directly (using its "environmental profiles" as a first step), so it could be considered as partly belonging to the fourth category, with an extra stage bringing it into the first category. However SHEBA certainly does not belong to the second or third categories since it does not perform matching at the SSE level. Note also that any method in the second category (SSE alignment) can be transformed into a method in the third category by adding to it some method of extending the SSE alignment to a residue alignment.

Some recent methods use information about SSE orientation to match proteins by global geometric information. TableauSearch and IR Tableau [17] make use of tableaux [18] to perform rapid and accurate comparison of whole protein structures. QP Tableau Search [19], another tableau-based method, is closer to the original quadratic integer programming (*QIP*) formulation defined by Konagurthu *et al. *[3] and (unlike the other methods mentioned previously) allows substructure (motif) searching and non-sequential matchings. The latter refers to sets of correspondences between SSEs in which the sequential order of corresponding SSEs is not preserved. It is, however, considerably slower than most other (non-alignment) methods. ProSMoS [4] also makes use of SSE orientations, but takes quite a different approach from most other methods. It does not compare structures against each other, but rather a query motif is defined by the user as a "meta-matrix", which is then used to find structures that contain a substructural motif which matches the query motif thus defined.

Methods that use SSEs require some method for assigning secondary structure to proteins. That is, some method to classify residues in the protein as belonging to helices (of various kinds) or strands (as part of a *β*-sheet), or not part of an SSE. This can be done by a variety of methods, such as pattern recognition of hydrogen bonds and geometrical features, as in probably the best-known method, DSSP [20], or by hydrogen bonds and statistically derived backbone torsional angle information as in STRIDE [21]. Assignment of secondary structure is not an exact procedure, with various methods disagreeing about the exact beginning and end of SSEs, and particular SSEs depart significantly from the "ideal" model of Pauling and Corey [22-24]. In addition, using only SSEs means that regions of protein structure not defined as being part of an SSE are not used at all, which can lead to less sensitive results. In some cases, this excludes a structure from being processed at all, if the method of assignment determines that it contains no SSEs. At least a partial solution to this can be to use a method of defining SSEs that assigns a larger fraction of the structure to SSEs, as is done by ProSMoS, which uses the PALSSE method [25], specifically designed for use in protein structure similarity searching.

Another protein structure comparison method is maximum contact map overlap (*MAX-CMO*), which consists of finding an alignment of residues in two proteins that maximizes the overlap between their contact maps. That is, it maximizes the number of contacts where residues that are in contact in one protein are aligned with residues that are also in contact in the other [26]. Since MAX-CMO operates at the level of residues, it belongs to the first of the four categories we have described.

MAX-CMO has been solved exactly by various methods including Lagrangian relaxation [27,28] and branch-and-bound [29], but these methods are often impractically slow and so heuristics have been used to approximate the solution [30]. However, compared to the MSVNS4MaxCMO heuristic [30], it has been shown that a tableau-based method is faster and has equal or greater accuracy in ranking protein similarity [19].

In this paper, we demonstrate SA Tableau Search, a new heuristic for tableau-based protein structural and substructure searching based on *simulated annealing *[31], and a parallel implementation of it on a modern general-purpose graphics processing unit (*GPGPU*). We compare its accuracy as a fold classification method with the existing methods DaliLite, SHEBA, VAST, YAKUSA, SARF2, LOCK2, TOPS, TableauSearch, QP Tableau Search, and IR Tableau on three different data sets: a set of 200 queries in the ASTRAL SCOP 1.75 95% sequence identity non-redundant database [32,33], all-against-all queries in the Fischer data set [34], and the COPS benchmark [35]. Because SA Tableau Search has a parameter, the number of *restarts *of its simulated annealing schedule, that can be adjusted as a tradeoff between speed and accuracy, we perform these comparisons with a number of different values of this parameter.

Three of the methods we compare SA Tableau Search with are also tableau-based methods: TableauSearch, QP Tableau Search, and IR Tableau. Although the tableau-based methods generally belong to the second category described above, since tableaux are defined in terms of SSEs, IR Tableau is an exception, belonging instead to the fourth class. By reducing the tableau representation to feature vectors which consist of counts of the number of different SSE orientation relationships in a structure's tableau, IR Tableau can compare structures by *cosine similarity*, that is, just the cosine of the angle between the feature vectors. This is extremely fast, but results in no alignment of SSEs, only a similarity score. TableauSearch is described in the original tableau-based protein structure searching paper as an approximation to the maximally-similar subtableaux extraction problem, which is an NP-hard problem [3]. It uses an alignment-like approach with two phases of dynamic programming, and is fast (but not as fast as IR Tableau), but is inherently sequential (it cannot find non-sequential structural matchings), and cannot find substructure matchings (motifs). Unlike IR Tableau, however, TableauSearch is capable of providing a set of correspondences between SSEs. QP Tableau Search is another method of approximating a solution to this problem, by relaxing the original QIP to a *quadratic program *(QP) [19]. This means that some of the desirable properties of the original (exact) formulation are retained, specifically that non-sequential and substructure matches can be found. In addition, QP Tableau Search introduces an additional constraint, that the difference in the distances between matched SSEs cannot exceed a threshold, which is found to increase accuracy when used as a protein structural database scanning method assessed on fold classification.

Of the methods described so far, only ProSMoS, TOPS, QP Tableau Search and SA Tableau Search are capable of substructure (motif) queries, and in fact ProSMoS is designed specifically to search for manually defined motifs in a database of structures, rather than structure similarity searching. In both these cases the motifs are defined at the SSE (not residue) level. None of the residue-based methods (such as DaliLite and SHEBA) are designed to allow a motif (substructure) query, as they assess structural similarity to find common regions between two structures and find an optimal superposition. As discussed in the description of ProSMoS [4], motifs, in contrast, are defined by "the main-chain topology, and general orientation and packing of secondary structural elements" [[4], p. 1331]. TOPS allows specification of such motifs, but operates on topological similarity only, while the QP Tableau Search and SA Tableau Search algorithms use tableaux, which are based on SSE orientation, and distance matrices. ProSMoS makes use of "meta-matrices" which include information on SSE orientation and contacts including hydrogen-bonding. Graphics processing units (*GPU*s) have recently been used for several bioinformatics applications, such as sequence alignment [36-39], molecular dynamics [40,41], microarray data analysis [42], mass spectrometry data analysis [43], and phylogenetics [44]. However, despite the large variety of protein structural search methods and their computationally intensive nature, to the best of our knowledge this is the first use of GPUs to accelerate protein structural or substructural searching.

## Results and Discussion

### Protein structure search (fold classification)

**Figure 1:**
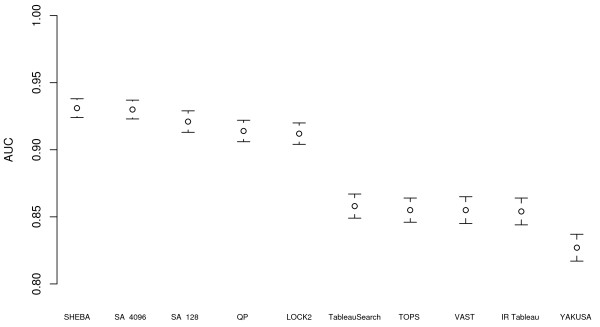
**AUC for different methods for 200 queries in the ASTRAL 95% data set**. Area under the ROC curve (AUC) for different methods on the 200 query set against the ASTRAL SCOP 95% sequence identity non-redundant database, excluding comparisons of a structure against itself. Each AUC value is shown with a 95% confidence interval. SA refers to SA Tableau Search (followed by number of restarts) and QP refers to QP Tableau Search. SARF2 is not present in this graph, as it is unable to process a data set this large, and DaliLite is not present as it exceeds the CPU time limit.

Figure 1 shows the *AUC *(area under the ROC curve -- see Methods section) and Table 1 shows AUC and elapsed time for several methods run on a set of 200 queries in the ASTRAL SCOP 1.75 95% sequence identity non-redundant subset. Run with 128 restarts, SA Tableau Search is one of the faster methods even on the host CPU, taking approximately the same time as YAKUSA: only TableauSearch, TOPS, and (especially) IR Tableau are faster. Run with 4096 restarts, SA Tableau Search is one of the most accurate methods, with AUC not statistically significantly different from that of SHEBA (Table 2), but considerably slower. Note that the results for SARF2 are not included for this data set as it is unable to process a data set this large, and DaliLite results are omitted, as it exceeds a CPU time limit of 400 hours.

**Table 1:** AUC and elapsed time for 200 queries in the ASTRAL 95% data set.

						95% confidence interval	
Method	Platform	Restarts	Elapsed time	AUC	Standard error	lower	upper
SHEBA	CPU	-	25 h 22 m	0.931	0.004	0.924	0.938
SA Tableau Search	CPU	4096	142 h 42 m	0.930	0.004	0.923	0.937
SA Tableau Search	GTX 285	4096	4 h 11 m	0.930	0.004	0.923	0.937
SA Tableau Search	Tesla C1060	4096	5 h 40 m	0.930	0.004	0.923	0.937
SA Tableau Search	CPU	128	4 h 18 m	0.921	0.004	0.913	0.929
SA Tableau Search	GTX 285	128	0 h 08 m	0.920	0.004	0.912	0.927
SA Tableau Search	Tesla C1060	128	0 h 11 m	0.920	0.004	0.912	0.927
QP Tableau Search	CPU	-	157 h 51 m	0.914	0.004	0.906	0.922
LOCK2	CPU	-	208 h 09 m	0.912	0.004	0.904	0.920
TableauSearch	CPU	-	1 h 11 m	0.858	0.005	0.848	0.867
TOPS	CPU	-	1 h 11 m	0.855	0.005	0.845	0.864
VAST	CPU	-	14 h 26 m	0.855	0.005	0.846	0.865
IR Tableau	CPU	-	0 h 01 m	0.854	0.005	0.844	0.864
YAKUSA	CPU	-	4 h 14 m	0.827	0.005	0.816	0.837

Area under the ROC curve (AUC) and elapsed time for different methods on the 200 query set against the ASTRAL SCOP 95% sequence identity non-redundant database. The table is sorted by AUC descending.

In the Platform column, either a GPU card is described, or CPU, which is a single core of an AMD Quad Core Opteron (2.3 GHz, 32 GB RAM) running Linux.

SARF2 is not present in this table, as it is unable to process a data set this large, and DaliLite is not present as it exceeds the CPU time limit.

**Table 2:** ΔAUC relative to SA Tableau Search for 200 queries in the ASTRAL 95% data set.

Method(s)	ΔAUC
SA Tableau Search 4096, SHEBA, SA Tableau Search 8192	0.000
SA Tableau Search 2048	0.0007
SA Tableau Search 1024	0.0012
SA Tableau Seach 512	0.0023
SA Tableau Search 256	0.0039
SA Tableau Search 128	0.0090
QP Tableau Search	0.0160
LOCK2	0.0183
TableauSearch	0.0723
VAST	0.0746
TOPS	0.0756
IR Tableau	0.0761
YAKUSA	0.1034

Difference in AUC relative to SA Tableau Search (4096 restarts) for different methods on the 200 query set against the ASTRAL SCOP 95% sequence identity non-redundant database. The table is sorted by ΔAUC, so that methods with lower AUC than SA Tableau Search (ΔAUC > 0, with p-value < 0.05) are at the bottom of the table. Methods for which there is no statistically significant difference in AUC from SA Tableau Search (4096 restarts) at p-value 0.05 are shown in a single row with ΔAUC = 0.000.

**Figure 2:**
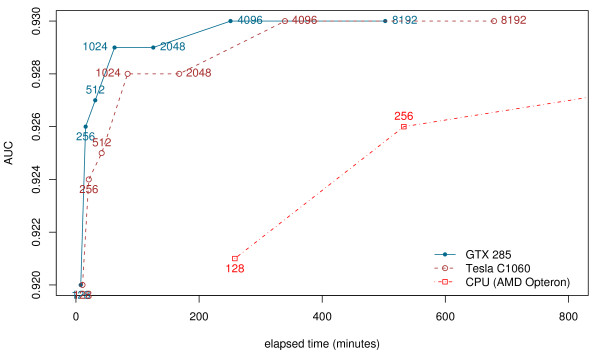
**AUC versus elapsed time for SA Tableau Search**. AUC versus elapsed time for SA Tableau Search on different platforms for the 200 query set against the ASTRAL SCOP 95% sequence identity non-redundant database. Each data point is an AUC value, and is labelled with the number of restarts that SA Tableau Search is run with.

Considering now the GPU implementation of SA Tableau Search, we can see that on average the GTX 285 card provides a 33 times speedup (34 times for 4096 restarts), and the Tesla C1060 card provides on average a 24 times speedup. SA Tableau Search run with 4096 restarts on the GTX 285 takes 4 hours and 11 minutes, making it not only one of the most accurate methods considered, but also one of the fastest. Figure 2 shows that, while elapsed time obviously increases linearly with the number of restarts (above 128, the number run in parallel), the accuracy as measured by the AUC figure increases much more slowly, and there is no significant difference between the value for 4096 and for 8192 restarts (Table 2). Hence, although changing the number of restarts allows a user-selectable tradeoff between accuracy and speed, one runs into rapidly diminishing returns for numbers of restarts beyond a certain point; on this evaluation data set, using more than 4096 restarts does not increase the AUC, and 512 restarts (taking therefore approximately 1/8 the time of 4096 restarts) is enough to achieve the same accuracy (within statistical significance at p-value 0.05) as SHEBA but in just 31 minutes 25 seconds on the GTX 285. On the host CPU, this takes 17 hours 53 minutes, and SHEBA takes over 25 hours.

**Figure 3:**
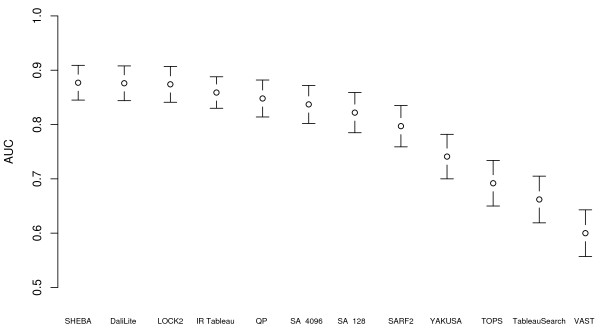
**AUC for different methods in the Fischer data set**. Area under the ROC curve (AUC) for different methods for all-against-all comparisons in the Fischer data set, excluding comparisons of a structure against itself. Each AUC value is shown with a 95% confidence interval. SA refers to SA Tableau Search (followed by number of restarts) and QP refers to QP Tableau Search.

Figure 3, Table 3, and Table 4 show the AUC and elapsed time for all-against-all queries in the Fischer data set. This data set is much smaller, but was constructed specifically to benchmark fold recognition and so contains structurally similar proteins with very low sequence similarity [34]. In this data set, QP Tableau Search, SHEBA, DaliLite and LOCK2 all have statistically significantly higher AUC values than SA Tableau Search, although the latter when run on a GPU card is considerably faster than these methods. We find that IR Tableau works particularly well on this data set, with an AUC not statistically significantly different from that of SA Tableau Search, and faster (on the host CPU) than even SA Tableau Search on the GTX 285 card (the fastest available to us).

**Table 3:** AUC and elapsed time in the Fischer data set.

						95% confidence interval	
Method	Platform	Restarts	Elapsed time	AUC	standard error	lower	upper
SHEBA	CPU	-	03 m 47 s	0.877	0.016	0.845	0.909
DaliLite	CPU	-	113 m 34 s	0.876	0.016	0.845	0.908
LOCK2	CPU	-	32 m 12 s	0.874	0.016	0.842	0.907
IR Tableau	CPU	-	< 1 s	0.859	0.015	0.830	0.888
QP Tableau Search	CPU	-	52 m 10 s	0.848	0.018	0.813	0.882
SA Tableau Search	CPU	4096	12 m 07 s	0.837	0.018	0.801	0.872
SA Tableau Search	Tesla C1060	4096	00 m 55 s	0.836	0.018	0.800	0.871
SA Tableau Search	GTX 285	4096	00 m 40 s	0.829	0.018	0.792	0.865
SA Tableau Search	CPU	128	00 m 25 s	0.822	0.019	0.786	0.859
SA Tableau Search	GTX 285	128	00 m 02 s	0.809	0.019	0.771	0.846
SA Tableau Search	Tesla C1060	128	00 m 02 s	0.804	0.019	0.767	0.842
SARF2	CPU	-	19 m 34 s	0.797	0.020	0.759	0.835
YAKUSA	CPU	-	00 m 02 s	0.741	0.021	0.700	0.782
TOPS	CPU	-	01 m 05 s	0.692	0.022	0.649	0.734
TableauSearch	CPU	-	< 1 s	0.662	0.022	0.619	0.705
VAST	CPU	-	02 m 33 s	0.600	0.022	0.557	0.643

Area under the ROC curve (AUC) and elapsed time for different methods for all-against-all comparisons in the Fischer data set. The table is sorted by AUC descending. In the Platform column, either a GPU card is described, or CPU, which is a single core of an AMD Quad Core Opteron (2.3 GHz, 32 GB RAM) running Linux.

**Table 4:** ΔAUC relative to SA Tableau Search in the Fischer data set.

Method(s)	ΔAUC
QP Tableau Search	-0.0533
SHEBA	-0.0409
DaliLite	-0.0399
LOCK2	-0.0379
SA Tableau Search 4096, IR Tableau	0.000
SA Tableau Search 1024	0.0055
SA Tableau Search 128	0.0143
SARF2	0.0398
YAKUSA	0.0954
TOPS	0.1449
TableauSearch	0.1744
VAST	0.2365

Difference in AUC relative to SA Tableau Search (4096 restarts) methods for all-against-all comparisons in the Fischer data set. The table is sorted by ΔAUC, so that methods with higher AUC than SA TableauSearch (ΔAUC < 0, with p-value < 0.05) are at the top of the table, and those with lower AUC than SA tableau search (ΔAUC > 0, with p-value < 0.05) are at the bottom of the table. Methods for which there is no statistically significant difference in AUC from SA Tableau Search (4096 restarts) at p-value 0.05 are shown in a single row with ΔAUC = 0.000.

**Figure 4:**
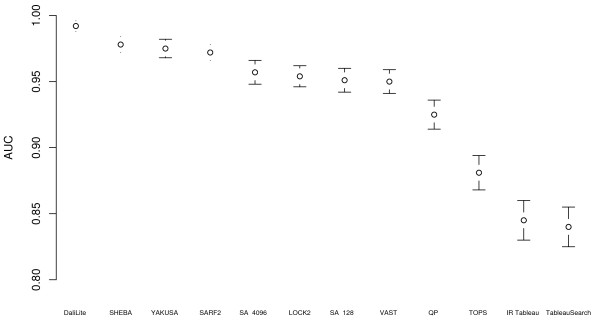
**AUC for different methods in the COPS benchmark data set**. Area under the ROC curve (AUC) for different methods for the COPS benchmark data set. Each AUC value is shown with a 95% confidence interval. SA refers to SA Tableau Search (followed by number of restarts) and QP refers to QP Tableau Search.

Figure 4, Table 5, and Table 6 show the AUC and elapsed time for the queries in the COPS benchmark [35]. DaliLite, SHEBA, YAKUSA, and SARF2 have the best AUC values for this data set, followed by SA Tableau Search which has an AUC value not significantly different from that of VAST and LOCK2. Although it is the best performing on AUC, DaliLite is by a considerable margin the slowest program. In this benchmark, TOPS and the other tableau based methods have significantly lower AUC than the other methods.

**Table 5:** AUC and elapsed time in COPS benchmark data set.

						95% confidence interval	
Method	Platform	Restarts	Elapsed time	AUC	standard error	lower	upper
DaliLite	CPU	-	123 h 29 m 47 s	0.992	0.002	0.989	0.996
SHEBA	CPU	-	6 h 04 m 16 s	0.978	0.003	0.971	0.984
YAKUSA	CPU	-	0 h 02 m 25 s	0.975	0.003	0.969	0.982
SARF2	CPU	-	13 h 57 m 34 s	0.972	0.004	0.964	0.978
SA Tableau Search	CPU	4096	8 h 41 m 18 s	0.957	0.004	0.949	0.966
SA Tableau Search	GTX 285	4096	0 h 26 m 59 s	0.957	0.004	0.949	0.966
SA Tableau Search	Tesla C1060	4096	0 h 30 m 30 s	0.957	0.004	0.948	0.965
LOCK2	CPU	-	72 h 11 m 06 s	0.954	0.005	0.945	0.962
SA Tableau Search	CPU	128	0 h 15 m 17 s	0.951	0.005	0.942	0.960
VAST	CPU	-	1 h 37 m 53 s	0.950	0.005	0.941	0.959
SA Tableau Search	Tesla C1060	128	0 h 03 m 00 s	0.948	0.005	0.939	0.958
SA Tableau Search	GTX 285	128	0 h 03 m 02 s	0.947	0.005	0.938	0.957
QP Tableau Search	CPU	-	74 h 47 m 43 s	0.925	0.006	0.914	0.936
TOPS	CPU	-	0 h 18 m 25 s	0.881	0.007	0.868	0.894
IR Tableau	CPU	-	< 1 s	0.845	0.008	0.831	0.860
TableauSearch	CPU	-	0 h 18 m 07 s	0.840	0.008	0.825	0.855

Area under the ROC curve (AUC) and elapsed time for different methods on the COPS benchmark data set. The table is sorted by AUC descending. In the Platform column, either a GPU card is described, or CPU, which is a single core of an AMD Quad Core Opteron (2.3 GHz, 32 GB RAM) running Linux.

**Table 6:** ΔAUC relative to SA Tableau Search in the COPS benchmark data set.

Method(s)	ΔAUC
DaliLite	- 0.0349
SHEBA	- 0.0201
YAKUSA	- 0.0180
SARF2	- 0.0140
SA Tableau Search 4096, VAST, LOCK2	0.000
SA Tableau Search 128	0.0064
QP Tableau Search	0.0325
TOPS	0.0764
IR Tableau	0.1120
TableauSearch	0.1172

Difference in AUC relative to SA Tableau Search (4096 restarts) methods on the COPS benchmark data set. The table is sorted by ΔAUC, so that methods with higher AUC than SA Tableau Search (ΔAUC < 0, with p-value < 0.05) are at the top of the table, and those with lower AUC than SA tableau search (ΔAUC > 0, with p-value < 0.05) are at the bottom of the table. Methods for which there is no statistically significant difference in AUC from SA Tableau Search (4096 restarts) at p-value 0.05 are shown in a single row with ΔAUC = 0.000.

The Fischer and COPS data sets have quite different properties. The Fischer data set was designed to benchmark fold recognition methods, independent of the structural similarity score being tested. That is, it was originally constructed to benchmark methods for assigning a sequence of unknown structure to a known fold. Part of this benchmark consists of an assignment of protein sequences to their most compatible fold, on purely structural criteria, independent of the protein representation and similarity scores used by the fold recognition methods to be assessed [34]. This benchmark has since been used, as we do here, for the somewhat different purpose of benchmarking methods that compare two known protein structures, for example in Pelta *et al. *[30]. No two proteins in the data set have sequence similarity over 35% [34], and it is quite small, consisting of only 68 structures. We perform all-against-all queries in this data set, resulting in 4624 pairwise comparisons. The COPS benchmark, in contrast, was designed to benchmark sequence similarity database searches, and true positives in this benchmark are defined by the COPS classification [35,45]. This classification is defined by structural similarity according to the TopMatch structural alignment algorithm [46,47], and so the COPS benchmark in fact assesses a method according to its agreement with TopMatch.

We note that in these last two benchmark data sets (Fischer and COPS), in both cases, four methods have a statistically significantly higher AUC than SA Tableau Search. However, it is a different set of four methods in the two cases, with SHEBA and DaliLite in common. Hence, given the different purposes of the Fischer and COPS benchmarks, it would seem that SHEBA and DaliLite are superior for both "difficult" (distantly related and low sequence similarity) and "easy" (more closely related) database searching tasks. YAKUSA and SARF2 perform better only on the more closely related searches (or, to be precise, agree more closely with TopMatch), and LOCK2 and QP Tableau Search perform better on the more distantly related searches.

In summary, over the three benchmark data sets we tested, no one method is consistently the best performing, although SHEBA is consistently in the top two methods measured by AUC, and it is considerably faster than DaliLite, the only method that has a higher accuracy on one of the benchmarks. All the other methods tested appear at different ranks in different benchmarks. SHEBA and SA Tableau Search are the top two ranking methods, with no statistically significant difference in AUC (at p-value 0.05), in the ASTRAL 95% 200 query benchmark. This data set is the largest one, but it also contains many similar structures and sequences. In all three benchmark data sets, SA Tableau Search has a (statistically significantly) higher AUC than TOPS and TableauSearch, and in all but COPS (where it has a not significantly different AUC), it has a higher AUC than VAST.

It is worth noting that QP Tableau Search and SA Tableau Search use exactly the same formulation of the protein substructure search problem as the extraction of maximally-similar subtableaux first described by Konagurthu *et al. *[3], enhanced by distance difference constraints introduced in Stivala *et al. *[19]. The difference is in the method of approximation: QP Tableau Search relaxes the problem to a QP to find a (locally) optimal solution, while SA Tableau Search, described here, uses simulated annealing. Not only is the latter method faster and often more accurate (with sufficient restarts) on the host CPU than QP Tableau Search, but its simplicity allows parallelization on GPU cards, which are currently quite restricted in some respects, such as availability of sophisticated math libraries. For example, the complexity of QP Tableau Search in its use of an interior point solver makes it impossible for us to implement a GPU version. As will be described in detail in the Methods section, there are two levels of parallelization in our implementation: first, each restart of the simulated annealing schedule for a single comparison of two structures is run in parallel, and second, multiple comparisons between a single query and multiple database structures are run in parallel. Any database scanning method can be trivially parallelized in the second way, since each pairwise comparison is independent; to implement this at all on a GPU requires that the method is capable of being implemented within the limited computational and fast memory resources of the GPU. Since there is no recursion and no dynamic memory allocation in the NVIDIA CUDA programming model [48], this can make implementing sophisticated algorithms such as those required for pattern searching in YAKUSA [12], for example, extremely difficult, if not impossible. Even where this is possible, since the GPU is essentially a data-parallel architecture [48], efficient implementations require that essentially the same code path is run simultaneously in a large number of threads, just with different data. The more threads diverge in their code path, the less efficient the parallelization will be, another reason why very simple algorithms are more suited to such parallelization. An efficient parallel implementation on a GPU requires in addition a "fine-grained" level of parallelization, in order to maximize the usage ("occupancy") of the thread multiprocessors [48] and the small amount of fast shared memory that this finer level of parallelization has access to. SA Tableau Search is ideally suited to this architecture since its data (tableaux and distance matrices) are shared between the threads running each restart of the simulated annealing schedule independently, and are usually small enough to fit in the fast shared memory. It may be possible to parallelize SHEBA in a similar manner. However, assuming a similar two-level parallelization, where the coarser level is to run multiple comparisons independently in parallel, and the finer level is to parallelize each comparison, this would require a parallelization of both the dynamic programming procedure and the three-dimensional translation and rotation procedure used iteratively by SHEBA [16]. This is a much more challenging task than simply running the multiple restarts of SA Tableau Search in parallel.

### Substructure queries

Evaluating the accuracy of substructure (motif) queries in a quantitative and objective way such as AUC is quite challenging; there is no database such as SCOP to provide a set of all true occurrences of a motif in general. We therefore provide two examples where we can provide such an evaluation: the *β*-grasp motif from ubiquitin [49], and the serpin (serine protease inhibitor) B/C sheet substructure [50]. We perform these queries in the ASTRAL SCOP 1.75 95% sequence identity non-redundant database.

**Figure 5:**
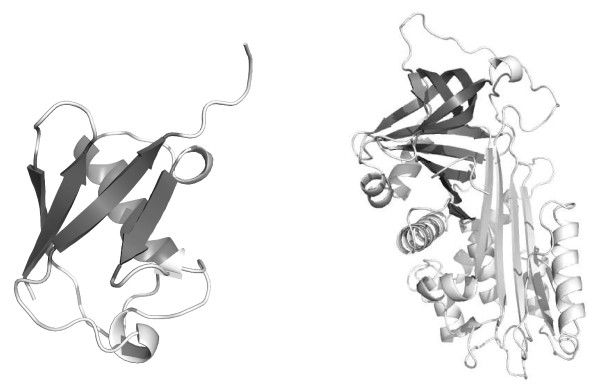
**substructure (motif) queries**. d1ubia_ (all SSEs) and *β*-grasp motif query (darker shaded SSEs only) structures (left), and 3D structure of the canonical active serpin, *α*_1_-antitrypsin, PDB id 1QLP, showing the B/C sheet used as the substructure query as the darker shaded SSEs (right). Images created with PyMOL [81].

In evaluating the accuracy of a substructure query for the *β*-grasp motif, we use the data from Table 1 of Shi *et al. *[4] as the gold standard. A hit is considered a true positive if it is in the same SCOP superfamily as the exemplars listed in Table 1 of Shi *et al. *[4] for the *β*-grasp core and gregarious fold [51] categories, or if it is one of the structures considered by Shi *et al. *[4] to contain the *β*-grasp motif by structural drift [52]. We demonstrate two queries for this test. First we use ubiquitin (SCOP identifier d1ubia_), an exemplar of the *β*-grasp fold, as the query. Second, we use a subset of the SSEs, namely the four largest strands and the *α*-helix in d1ubia_, chosen to represent the essential part of the *β*-grasp motif. This motif query is based on that defined by Shi *et al. *[4] as a "meta-matrix" for ProSMoS. These query structures are illustrated in Figure 5.

The serpin B/C sheet substructure (see Figure 5) is such a large and specific structure that we can be confident it is truly present only in instances of the serpin fold. Therefore, in evaluating the accuracy of a substructure query for this substructure, we consider a hit a true positive if it is a member of the serpin fold in SCOP, and a false positive otherwise. We choose the B/C sheet of the canonical active serpin, *α*_1_-antitrypsin, PDB id 1QLP[53] as the query structure.

Of the protein structure comparison methods benchmarked in this paper, only QP Tableau Search, SA Tableau Search, TOPS and ProSMoS are also substructure (motif) finding methods. However, comparison using AUC with ProSMoS is not possible as ProSMoS does not rank all database structures. Instead, it returns a set of structures that contain the query motif. The web server version [54] provides a ranking within this set only, the downloadable version provides no scores or ranking.

Table 7 shows the results of these substructure queries. We can see that, compared to QP Tableau Search, SA Tableau Search has similar accuracy, and is up to 6 times faster on the CPU implementation. The GPU implementation (on the GTX 285 card) is faster still, providing a speedup of up to 26 times over the CPU implementation of the same algorithm.

**Table 7:** AUC and elapsed time for some motif (substructure) queries.

							95% confidence interval	
Query	Method	Platform	Restarts	Elapsed time	AUC	standard error	lower	upper
d1ubia_	SA	GTX 285	128	00 m 03 s	0.918	0.011	0.896	0.940
d1ubia_	SA	CPU	128	01 m 17 s	0.912	0.011	0.890	0.935
d1ubia_	QP	CPU	-	05 m 11 s	0.902	0.012	0.879	0.926
d1ubia_	TOPS	CPU	-	00 m 10 s	0.894	0.012	0.870	0.918

*β*-grasp	SA	CPU	128	01 m 11 s	0.939	0.010	0.920	0.958
*β*-grasp	QP	CPU	-	02 m 01 s	0.938	0.010	0.918	0.957
*β*-grasp	SA	GTX 285	128	00 m 03 s	0.934	0.010	0.914	0.954
*β*-grasp	TOPS	CPU	-	00 m 09 s	0.847	0.014	0.819	0.875

serpin B/C sheet	SA	GTX 285	128	00 m 03 s	0.993	0.013	0.968	1.019
serpin B/C sheet	SA	CPU	128	01 m 19 s	0.991	0.015	0.962	1.021
serpin B/C sheet	QP	CPU	-	08 m 16 s	0.986	0.019	0.949	1.023
serpin B/C sheet	TOPS	CPU	-	00 m 24 s	0.491	0.054	0.385	0.597

Area under the ROC curve (AUC) and elapsed time for some motif (substructure) queries on the ASTRAL SCOP 95% sequence identity non-redundant database. In the Method column, QP refers to the QP Tableau Search method, SA refers to SA Tableau Search. In the Platform column, either a GPU card is described, or CPU, which is a single core of an AMD Quad Core Opteron (2.3 GHz, 32 GB RAM) running Linux. The results for each query are sorted by AUC descending.

It should be noted that (as described in the Methods section), d1ubia_ is one of the queries used in tuning the simulated annealing parameters. For that purpose it was evaluated as a fold recognition task, rather than as a substructure query. Hence SA Tableau Search could not be expected to perform badly on the d1ubia_ query as it has been in some part optimized for this query. This is not the case, however, for the serpin B/C sheet substructure.

## Conclusions

We have demonstrated a simulated annealing heuristic for tableau-based protein structure and substructure searching, that is as fast or faster, and comparable in accuracy, with some widely used existing methods when run on a standard CPU. In addition, we have provided a parallel implementation on modern GPU cards that achieves a speedup of up to 34 times over the CPU implementation, making it one of the fastest available methods. To the best of our knowledge, this is the first application of GPUs to the protein structural search problem.

There may well be scope for application of this technique to other optimization problems in bioinformatics that can be approximated by relatively simple optimization heuristics capable of being parallelized by implementation on a GPU. For example, the multistart variable neighborhood search (VNS) heuristic for maximum contact map overlap [30], or some other heuristic such as simulated annealing for the same problem, could benefit from parallelization on the GPU. Another candidate problem of much current interest is biological network alignment, a highly computationally intensive problem that has previously been solved by quadratic programming [55], and has recently been approximated by local search heuristics [56] amongst other methods.

## Methods

### Tableaux and distance matrices

A tableau is a discrete encoding of the orientation matrix for a protein structure, originally defined by Lesk [18]. Other work has shown that tableaux can accurately differentiate folds [57], and can be used for rapid protein structural comparison [3,17] and for protein substructural search [19].

The orientation matrix is a square symmetric matrix describing the relative orientation of secondary structure elements (SSEs) in the protein. Each element *ω_ij_*, 1 ≤ *i*, *j *≤ *N *of the orientation matrix for a structure with *N *SSEs is the relative angle between SSEs *i *and *j*, numbered from the N- to the C-terminus. This matrix is computed by fitting axes to each SSE, and, for every pair of SSEs, computing the relative angles between their axes. This interaxial angle is defined as the smallest angle required to reorient one axis vector so that it eclipses the other, around the mutual perpendicular between the two vectors; or, equivalently, the angle between the two vectors projected onto a plane normal to their mutual perpendicular [3].

**Figure 6:**
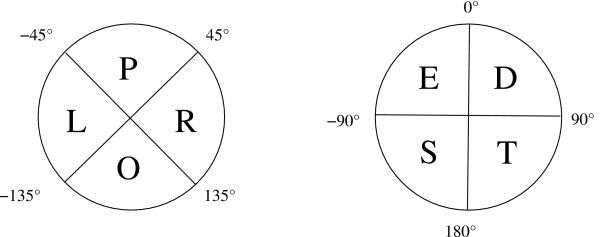
**Double-quadrant encoding of orientations**. The relative orientation of any two SSEs is encoded as a two-character string with the two quadrant schemes shown, which differ in orientation by 45° in order to prevent small variations resulting in completely different encodings. The first quadrant encoding is labelled P, O, L, R for parallel, anti-parallel, crossing-left, and crossing-right, respectively, and the second arbitrarily E, D, S, T[18]. For example, two SSEs that are anti-parallel would be encoded as either OS or OT.

The tableau is derived from the orientation matrix by means of a double-quadrant encoding scheme whereby the angles are classified into quadrants in two different ways that differ in orientation by *π*/4. This prevents a small variation in angle resulting in two completely different encodings [18], illustrated in Figure 6. For example, consider two SSEs that are anti-parallel; they may have an interaxial angle of, say, 143° The first part of the encoding scheme gives the tableau code as O (anti-parallel), and the second part as T, resulting in a tableau code OT for this angle.

**Figure 7:**
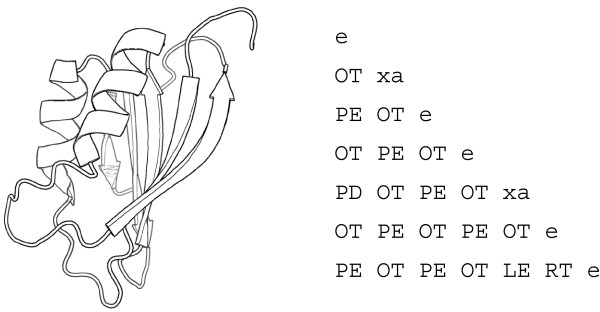
**Cartoon (left) and tableau (right) for acylphosphatase, PDB identifier **1APS. The main diagonal denotes the SSE type by e, xa, xi, or xg for *β*-strands, *α*-helices, *π*-helices, and 3_10_-helices, respectively. The cartoon was created with PyMOL [81].

We will denote the *N *× *N *tableau for a structure with *N *SSEs by *T *, with elements *t_ij _*, 1 ≤ *i*, *j *≤ *N *being tableau codes such as PE or OS. Because the tableau is symmetric, only the bottom triangle is stored, and since the main diagonal is redundant (the angle between an SSE and itself), it is used to store the type of the SSE for that row and column. Consider the two helices in Figure 7: they are anti-parallel, and have the tableau code OT. This can be seen by finding the two helix codes (xa) on the main diagonal. The helices in this structure are the 2nd and 5th SSEs, so the angles relative to these two SSEs are in row (and column) 2 and 5 of the tableau. Hence the entry for the angle between these two helices is their common row and column, which is OT. In fact, they have an interaxial angle of approximately 143°.

For a structure with *N *SSEs, the distance matrix is a square symmetric matrix *D *= (*d_ij_*), 1 ≤ *i*,*j *≤ *N *where each element is the distance (in Ångströms) between the centroids of the C_*α *_atoms in SSEs *i *and *j*. As with tableaux, these distance matrices apply to SSEs, not residues.

As originally defined by Lesk [18], and Kamat and Lesk [57], tableaux only contain entries for SSEs that are "in contact" with each other, that is, the corresponding entry in the distance matrix is below some threshold. In this paper, as in Konagurthu *et al. *[3] and Stivala *et al. *[19], we use a version of tableaux in which every entry has a value, regardless of the distance between the two SSEs.

### The tableau matching problem

The problem of finding a common (maximally-similar) substructure between two protein structures was formulated as a quadratic integer problem (QIP) of extracting maximally-similar subtableaux by Konagurthu *et al. *[3]. We use the same formulation here, using only the discrete tableau, not the continuous orientation matrix, and, as in our previous work [19], we also incorporate a distance matrix difference constraint (Equation 7).

Define Boolean variables *x_ij _*, 1 ≤ *i *≤ *N_A_*, 1 ≤ *j *≤ *N_B _*where *x_ij _*= 1 indicates that the *i*th SSE in structure A is matched with the *j*th SSE in structure B. Let $T_{A}=(t_{ij}^{A})$ and $T_{B}=(t_{ij}^{B})$ be tableaux for protein structures A and B with *N_A _*and *N_B _*SSEs, respectively. Define a scoring function as:

(1) $$\zeta (t_{ik}^{A},t_{jl}^{B})=\{\begin{array}{cc} 2, & \text{if }t_{ik}^{A}\equiv t_{jl}^{B}\\ 1, & \text{if }t_{ik}^{A}≃t_{jl}^{B}\\ -2, & \text{otherwise}. \end{array}$$

where $t_{ik}^{A}\equiv t_{jl}^{B}$ means the two tableau codes are identical, and $t_{ik}^{A}≃t_{jl}^{B}$ means they differ in only one quadrant.

Then the QIP is:

maximize

(2) $$f(x)=\sum_{{}_{1\leq i,k\leq N_{A},1\leq j,l\leq N_{B}}}\zeta (t_{ik}^{A},t_{jl}^{B})x_{ij^{X}kl}$$

subject to

(3) $$\begin{array}{cc} \sum_{j=1}^{N_{B}}x_{ij}\leq 1, & 1\leq i\leq N_{A} \end{array}$$

(4) $$\begin{array}{cc} \sum_{i=1}^{N_{A}}x_{ij}\leq 1, & 1\leq j\leq N_{B} \end{array}$$

Constraints (3) and (4) ensure that each SSE in one tableau is matched with at most one SSE in the other. We introduce a further condition: that two SSEs of different types (for example an *α*-helix and a *β*-strand) should not be matched, for which we use the SSE type information encoded on the diagonal of the tableau. We may optionally avoid non-sequential matchings by forbidding matches between SSEs whose indices *i*, *k *in one structure and *j*, *l *in the other satisfy both of the following inequalities:

(5) $$1\leq i<k\leq N_{A}$$

(6) $$1\leq l<j\leq N_{B}$$

Without this condition, non-sequential matchings can be found.

In order to avoid false positives when SSEs in two structures have similar orientations relative to other SSEs in their respective structures, but are at very different distances from those other SSEs, we use a distance difference constraint [19], disallowing matches between SSEs where the difference in distances between the SSEs exceeds a threshold distance *τ *:

(7) $$\begin{array}{cc} x_{ij}+x_{kl}\leq 1\;\;\text{if}\;\;|d_{ik}^{A}-d_{jl}^{B}|>\tau , & \begin{array}{c} 1\leq \;i,\;k\;\leq N_{A}\\ 1\leq \;j,\;l\;\leq N_{B} \end{array} \end{array}$$

where $D^{A}=(d_{ik}^{A}),\;1\leq i,\;k\leq N_{A}$ and $D^{B}=(d_{jl}^{B}),\;1\leq j,\;l\leq N_{B}$ are SSE midpoint distance matrices. We show here a heuristic for approximating an optimal solution of this problem that retains the capability, like QP Tableau Search, of finding substructure and non-sequential matchings, but is as fast or faster than many existing methods, and is capable of parallelization on appropriate hardware for even greater speed.

We use the well-known and relatively simple technique of simulated annealing, whereby the system has a global "temperature" that controls the probability of accepting a state change that does not improve the current objective function value. This helps prevent the system from becoming trapped in a local maximum. The temperature is decreased as the simulation progresses, so that such non-improving moves become less likely over time.

### CUDA

Modern graphics processing units (GPUs), often referred to as general-purpose GPUs (GPGPUs) in the context of applications other than graphics rendering, are highly parallel multithreaded processors, which can greatly accelerate data-parallel applications. We use here the NVIDIA CUDA programming model [48], which provides extensions to the C programming language in order to make use of NVIDIA GPUs. The CUDA model allows C functions called *kernels *to run in parallel on the GPU as *threads*. The threads are arranged in a hierarchy such that a fixed number of threads makes up a *block *of threads, and in turn a fixed number of blocks makes up a *grid*. Each block has a small (16 KB on the cards used in this paper) amount of shared memory, accessible by threads in that block only, which is as fast as register access. In contrast, the (large) global memory of the GPU, accessible by all threads, has very high latency. The *local *memory for each thread, not shared by any other, also has high latency. There is also a (small) constant memory, which all threads can read with low latency. It is therefore important to optimize the memory access pattern of kernels, and make the best use of the limited shared memory [48,58].

### Application of simulated annealing to the problem

Each structure in the database to be searched, and each query, is represented by its tableau and distance matrix. These tableaux and distance matrices are generated in advance.

We now describe the application of simulated annealing to the problem of extracting maximally-similar subtableaux.

The state (or configuration) of the system for matching structure *A *with *N_A _*SSEs and structure *B *with *N_B _*SSEs, represented by tableaux *T_A _*and *T_B _*, respectively, is represented by a vector *v *of dimension *N_A_*, where *v_i _*= *j*, 1 ≤ *i *≤ *N_A_*, 0 ≤ *j *≤ *N_B _*indicates that the *i*th SSE in structure A is matched with the *j*th SSE in structure B, or, if *v_i _*= 0, that the *i*th SSE in A is not matched with any SSE in B. The nonzero elements of *v*, regarded as a set, are constrained to be a subset of {1,..., *N_B_*}, so that each SSE in a structure is matched with at most one SSE in the other. This is achieved by constraints (3) and (4) in the QIP formulation. We can (optionally) forbid non-sequential matchings by ensuring that the nonzero elements of *v*, considered as a sequence, are strictly increasing, that is, *i *<*k *⇒ *v_k _*= 0 ∨ *v_i _*<*v_k_*, 1 ≤ *i, k *≤ *N_A_*.

Then, also incorporating the distance difference constraint, the objective function *g(v)*, that we seek to maximize, becomes

(8) $$\begin{array}{cc} g\left(v\right)= & \sum_{\begin{array}{l} 1\leq i,k\leq N_{A}\\ 1\leq j,l\leq N_{B} \end{array}}\{\begin{array}{ll} \zeta \left(t_{ik}^{A},t_{jl}^{B}\right)\;, & {\text{if}}_{\;\;\;\;\left|d_{ik}^{A}-d_{jl}^{B}\right|\leq \tau }^{\;\;\;v_{i}=j\wedge v_{k}=l\;\wedge }\\ 0, & \text{otherwise} \end{array} \end{array}$$

Note that this can then be computed efficiently, in $\text{O}(N_{A}^{2})$ time, by a nested iteration over the *v *vector, since the summand is nonzero only when *v_i _*= *j *Λ *v_k _*= l, that is, we need only consider values of *j *and *l *that are actually present in the *v *vector.

We set a random initial state of the system by matching, with probability *p_m_*, each SSE in structure A with the first SSE of the same type (helix or strand) in structure B, in sequence from 1 ... *N_A _*and 1 ... *N_B _*. At each iteration, the "move" to a neighbor state is generated by choosing uniformly at random an SSE, *i*, in structure A and changing its mapping to a random SSE, *j*, in structure B, from the set of such SSEs that satisfy the type and (optionally) ordering constraints on the mapping, giving a new state *v' *If no SSE in structure B that meets the constraints can be found, the SSE that was chosen is removed from the mapping, that is we set ${v^{\prime }}_{i}=0$.

The simulated annealing algorithm proceeds by efficiently computing the new value of the objective function (Equation 8) and accepting the new state if the new value of the objective g(*v'*) is greater than the maximum so far found, or if $\exp(\frac{g\left(v'\right)-g\left(v\right)}{T})>p$ where *p *is a random number in [0,1] and *T *is the current temperature of the system. The temperature is multiplied by the constant *α *for the next iteration. After the maximum number of iterations is reached, the state with the maximum value of the objective function found is returned as the best state.

The recomputation for a new state of the value of the objective function is computed in only O(*N_A_*) time by computing the difference in the value caused by the new SSE matching, rather than directly recomputing Equation 8. That is, the contribution to the score of the previous matching of the SSE *i *that was chosen to be changed is subtracted, and the contribution of its new mapping (or 0, if it is removed from the mapping) is added.

We optimize the simulated annealing parameters by using the eight folds in Table 1 of Stivala *et al. *[19] as queries against the ASTRAL SCOP 1.75 95% sequence identity non-redundant database as a training set, manually adjusting them to maximize the average AUC of these queries. We find that suitable values for these parameters are initial temperature *T_0 _*= 10, temperature multiplier *α *= 0.95, number of iterations 100, and initial SSE matching probability *p_m _*= 0.5. In addition, the entire simulated annealing process is run *M *times (by default, *M *= 128), and the best solution over all runs is returned. We use the same value *τ *= 4.0Å for the distance difference threshold as in Stivala *et al. *[19].

Tableau search implementations produce unnormalized scores, namely, a (locally) optimal value of the tableau scoring function. For comparing sets of pairwise scores between proteins of different sizes, a normalization function is required. We use the normalization function norm2 from Pelta *et al. *[30], which we found in our previous work [19] to be the best of the three normalization functions defined there:

(9) $$\text{norm}2(P_{i},P_{j})=2\cdot \frac{\text{score}(P_{i},P_{j})}{\text{size}(P_{i})+\text{size}(P_{j})}$$

where score is the tableau matching score and size is the number of SSEs (tableau dimension).

### Parallel implementation on a GPU

As well as its simplicity, another advantage of a simulated annealing heuristic such as that just described is that it is easily parallelizable. We run the *M *restarts of the simulated annealing schedule in parallel rather than serially. In addition, since in a structural database search the query is compared to many structures in the database, another level of parallelization is to compare the query to many database structures simultaneously. This two-level parallelization maps well to the CUDA programming model: each thread in a block of *B *threads runs the simulated annealing schedule, each from an independent random initialization, for a single comparison of the query to a database structure, and the grid of *G *blocks therefore executes *G *such comparisons simultaneously. That is, the query structure is compared to *G *database structures in parallel. If *M > B *then the *B *parallel executions of the simulated annealing process are repeated until all *M *restarts of the simulated annealing schedule have been run.

The entire database of tableaux and distance matrices is loaded into the (large) global memory, and the query structure (tableau and distance matrix) is loaded into the constant memory where it can be quickly read by all threads. Each block of *B *threads first parallel copies (that is, each thread copies one or more memory locations in parallel) the database tableau and distance matrix into shared memory for fast access during the actual simulated annealing process. The *G *blocks in the grid perform comparison of the query against *G *database structures simultaneously. For databases with more than *G *structures, this process is repeated until the whole database has been processed. By using the CUDA occupancy calculator [[58], Ch. 4] and some experimentation, we determine good values for *G *and *B *of 128 (for both), that is, a total of 16384 threads.

Because the size of the constant and shared memory is so small (16 KB on the graphics cards we used for this paper), structures in which the combined size of the tableau and distance matrix exceed this limit present a problem. We solve this problem by compiling two versions of the kernel, one of which uses shared memory for database structures and constant memory for query structures, and the other which leaves them both in the global memory. The shared memory is still used for the vector of maximum scores found in each thread, and the vector of SSE types (helix, strand) copied from the main diagonal of the tableau. The latter kernel is slower than the kernel using shared and constant memory, but removes any limitation on the size of structures that can be processed. A third version of the kernel is compiled for execution on the host CPU, running the same code as the kernel but without any GPU extensions and purely single-threaded.

An alternative solution is to process the structures too large for the shared memory on the host CPU simultaneously with the GPU processing the smaller structures. However we find that it is faster to run the two kernels serially on the GPU, even though of 16602 tableaux and distance matrices in the ASTRAL SCOP 1.75 95% sequence identity non-redundant database, only 668 are too large for the shared memory. Because the CUDA library does not contain an equivalent of the standard C library function rand() or similar, we use the CUDA SDK implementation [59] of the Mersenne Twister [60] pseudorandom number generator. We use the dynamic creation program [61] to create parameters to enable 16384 different Mersenne Twister pseudorandom number generators (one for each of the maximum number of threads we use on the GPU). Because the use of double precision floating point causes a considerable performance penalty on the GPU [58], we use only single precision.

An additional technique we use to increase the speedup on the GPU is to presort the tableau and distance matrix database by size, so that structures of similar size are processed simultaneously, thereby ensuring that the running times of blocks in the grid are as close as possible. A similar technique was used successfully by Manavski and Valle [37] for their CUDA acceleration of the Smith-Waterman [62] algorithm.

### Evaluation

We compute tableaux and distance matrices for all 16712 domains in the 95% sequence identity non-redundant subset of the ASTRAL SCOP 1.75 database [32,33]. This results in 16602 structures in our database, since 110 are omitted as DSSP [20] finds no SSEs for them.

We define a set of 200 queries chosen from the ASTRAL SCOP 1.75 95% sequence identity non-redundant data set. The queries are chosen at random, so that each class (*α*, *β*, *α/β*, *α + β*) is represented in the query set in the same ratio as it is in the database. This set is based on that defined in Stivala *et al. *[19] for ASTRAL SCOP 1.73; 14 structures in that set are no longer present in ASTRAL SCOP 1.75 and are replaced by others in the same superfamily. The list of queries is available with the source code and other data as described in the Availability section.

The Fischer data set, described in Table 2 of [34], consists of 68 proteins, from the classes *α*, *α/β*, *β*, *α + β*, and "other" (mixed *α *and *β*, and small proteins). We choose to benchmark on this data set as it contains proteins of very low sequence similarity and was constructed specifically to benchmark fold recognition methods, although it has since been used to benchmark protein structural comparison methods as well (for example in Pelta *et al. *[30]). All the major super families are included in the benchmark [34]. Several PDB identifiers in this table have since been obsoleted, and we replace these with their new versions according to the RCSB PDB website [63,64]. We perform an all-against-all comparison in this data set.

The COPS Benchmark 2009/6 data set [35] consists of a database of 1056 structures and a query set of 176 structures. The queries are not present in the database, and each query has exactly six true positives, structurally similar according to the COPS classification [45], but lacking a high degree of sequence similarity. This benchmark is designed to benchmark sequence similarity database searches, and true positives in this benchmark are defined by structural similarity according to the TopMatch structural alignment algorithm [46,47].

Comparisons of a query against itself are excluded from the results. Note that this can only occur where the query structures are also present in the database, as in the ASTRAL 95% 200 query set and the Fischer data set. It does not occur in the COPS benchmark, where the query structures are not present in the database. Comparisons for which a method can provide no score are assigned an arbitrary value which is at least as low as the lowest provided score for that method.

In the ASTRAL 95% 200 query set, we evaluate the accuracy of structural search by counting a hit (a score above the threshold) as a true positive if the structure is in the same SCOP fold as the query structure, and a false positive otherwise. For the Fischer data set, a true positive is counted when the score is above the current cutoff and the two structures are in the same fold according to Table 2 of [34]. For the COPS benchmark data set, the true positives are defined by COPS as described above.

For substructure search, true positives are defined by Table 1 of Shi *et al. *[4] for the *β*-grasp query, and by counting a hit as true positive only when it is to a structure in the "Serpins" fold according to SCOP for the serpin B/C sheet query.

We can then compute the true positive rate (TPR), or sensitivity, as $TPR=\frac{TP}{N}$ where *TP *is the number of true positives and *N *is the number of structures that match the query according to the gold standard. The false positive rate (FPR), which is equal to 1 - specificity, is $FPR=\frac{FP}{TN+FP}$ where *FP *is the number of false positives and *TN *is the number of true negatives. We then construct a ROC curve by plotting the *TPR *against the *FPR *for all values of the score threshold. The area under the ROC curve (*AUC*) is an overall measure of the quality of a classification method; a perfect classifier has *AUC *= 1.0, and a random classifier has *AUC *= 0.5.

When multiple queries, such as the 200 query set, are being evaluated in one ROC curve, all the scores are combined together (after normalization, if required), with each labelled as either a positive or negative according to the appropriate gold standard. That is, each individual query has a list of scores, one for each entry in the database. Each one of these entries is either positive or negative according to the relevant gold standard, that is, each individual database entry either is, or is not, in the same fold as the query. So each query has a set of tuples (*s*, *P*) where *s *is the score for that pairwise comparison and *P *is a Boolean variable that is *True *if the query and that database entry are in the same fold according to the gold standard for the database being used, and otherwise *False*. Each of these sets of tuples (one for each query) are then all combined into one large set. The ROCR package [65] in R [66] is then used to plot ROC curves and compute the Area Under the ROC Curve (AUC). 95% confidence intervals for the AUC values are calculated by the Hanley-McNeil method [67]. The statistical significance of ΔAUC values at p-value 0.05 are calculated with a nonparametric approach [68] using the StAR program [69].

### Implementation

We use the CUDA C compiler nvcc version 2.3, CUDA SDK version 2.3, and GNU C compiler gcc version 4.3.4. Host code is run on an AMD Quad Core Opteron (2.3 GHz, 2 processors, 32 GB RAM) running Linux. We use two GPU cards: an NVIDIA Tesla C1060 (4 GB global memory, 30 multiprocessors, 240 cores, 1.30 GHz) and an NVIDIA GeForce GTX 285 (1 GB global memory, 30 multiprocessors, 240 cores, 1.48 GHz). Both run the CUDA Driver and Runtime version 2.30. Scripts for generating tableaux and distance matrices and building the database, evaluating results against SCOP, and processing output for visualization are written in Python. We use the BioPython library [70] and the Bio.PDB file parsing and structure class [71] to parse PDB files and the Bio.SCOP interface [72] to read SCOP and ASTRAL data. For comparisons with other methods, SHEBA version 3.1.1, VAST downloaded from [73], YAKUSA downloaded from [74], the TOPS matching software downloaded from [75], DaliLite version 2.4.5, LOCK2 [10] downloaded from [76], and SARF2 [11] downloaded from [77] are used. We build the TOPS database for the ASTRAL SCOP 1.75 95% sequence identity non-redundant subset using TOPS downloaded from [78] (July 2007). A version of QP Tableau Search which uses the MA57 sparse symmetric solver [79,80] is used; this is considerably faster than the original implementation. The QP Tableau Search implementation is compiled with the Intel Fortran compiler (version 11.0). Default parameters are used for all programs, except that the -n option is used on YAKUSA to output a score for all database structures rather than just the top 50 hits. In computing the ROC curve, YAKUSA and DaliLite results are ranked by their Z-scores, SHEBA results by the SHEBA *m *value, LOCK2 results by native LOCK2 score, VAST results by *Pcli *value, TOPS results by native ("compression") score, and IR Tableau results by cosine similarity score. Normalization is only required for SA Tableau Search, QP Tableau Search and TableauSearch; results from these methods are ranked according to their scores normalized by Equation 9. SARF2 does not provide a matching score as such, but rather the number of residues aligned and the RMSD of the aligned residues. To combine these into a matching score, we use the SSM Q score [9]:

(10) $$Q=\frac{N_{align}^{2}}{(1+{\left(\text{RMSD}/R_{0}\right)}^{2})N_{1}N_{2}}$$

where *N_align _*is the number of residues aligned, RMSD is the root mean square deviation (in Ångströms) between these residues, and *R_0 _*= 3.0Å is an empirical parameter taken from [9].

We re-implement the IR Tableau [17] algorithm in Fortran 77, and compile it with the same compiler as QP Tableau Search.

For substructure (motif) queries with TOPS, the motif queries are constructed by editing the TOPS cartoons with the EditTops program and manually editing the resulting TOPS string.

## Availability and Requirements

• Project name: SA Tableau Search.

• Project home page: http://www.csse.unimelb.edu.au/~astivala/satabsearch

• Operating system(s): Linux, with NVIDIA CUDA Driver and Runtime version 2.30.

• Programming language: C, with NVIDIA CUDA extensions using the CUDA C compiler nvcc version 2.3 and CUDA SDK version 2.3, and GNU C compiler gcc version 4.3.4.

• Licence: None. This software can be used freely for any purpose.

• Any restrictions to use by non-academics: None.

## Authors' contributions

All authors contributed to the algorithm and evaluation design. AS implemented the algorithm and evaluation software, performed the tests, and prepared the manuscript and figures. All authors read and approved the final manuscript.
